# Temporal Neuroimmune Changes During Primary and Persistent Experimental Toxoplasmic Encephalitis

**DOI:** 10.3390/ijms27198577

**Published:** 2026-09-25

**Authors:** Gungor Cagdas Dincel, Hasan Tarik Atmaca

**Affiliations:** 1 Department of Medical Pathology, Faculty of Medicine, Ankara Medipol University, Ankara 06050, Türkiye; 2 College of Veterinary Medicine at Orange Park, Lincoln Memorial University, Orange Park, FL 32073, USA

**Keywords:** *Toxoplasma gondii*, toxoplasmic encephalitis, neuroimmunopathology, CD68, S100B, Foxp3, interleukin-5

## Abstract

*Toxoplasma gondii* persists in the central nervous system and may cause severe toxoplasmic encephalitis, particularly in immunocompromised hosts. Our previous studies demonstrated that *T. gondii*-induced neuropathology involves oxidative and nitrosative stress, apoptosis, glial activation, blood–brain barrier injury, and GMF-β- and SAP/NF-κB-associated responses. This study investigated the temporal immunoreactivity of CD68, S100B, Foxp3, and IL-5 during early and chronic experimental infection. Twenty-four female Swiss albino mice were assigned to one uninfected control group and three ME49-infected groups examined at 10, 30, and 60 days post-infection (*n* = 6/group). Marker immunoreactivity was evaluated by immunohistochemistry and quantitative image analysis. CD68 immunoreactivity was markedly increased at day 10, rose further by day 30, and remained high at day 60. In contrast, immunoreactivity for S100B, Foxp3, and IL-5 was limited at day 10 but increased markedly at days 30 and 60. Most notably, IL-5 continued to increase significantly from day 30 to day 60, whereas CD68, S100B, and Foxp3 remained at similarly high levels during this interval. These temporally divergent patterns demonstrate an early myeloid/phagolysosomal response followed by established astroglial, Foxp3-associated regulatory, and cytokine-associated tissue responses. The continued increase in IL-5, together with persistent neuropathological changes, supports the interpretation that late chronic toxoplasmic encephalitis is an active rather than quiescent neuroimmunopathological phase.

## 1. Introduction

*Toxoplasma gondii* (*T. gondii*) is one of the most widespread zoonotic protozoan parasites, infecting a broad range of warm-blooded hosts, including humans. Human infection occurs mainly through ingestion of tissue cysts in raw or undercooked meat, ingestion of environmentally resistant oocysts contaminating food or water, and congenital transmission [1]. Recent global analyses estimate that approximately one-third of the human population has been exposed to *T. gondii* [2]. Although infection is often asymptomatic in immunocompetent individuals, toxoplasmosis remains an important public health concern because of its widespread distribution, lifelong persistence, and potential to cause severe congenital, ocular, and neurological disease in susceptible populations [1].

During acute infection, *Toxoplasma gondii* disseminates in its tachyzoite form and can enter the central nervous system through direct passage across the blood–brain barrier or transport within infected leukocytes, initiating interactions with resident brain cells that contribute to neuroinflammation [3,4,5]. Following the acute phase, some tachyzoites differentiate into bradyzoites and form tissue cysts, particularly in neurons, thereby establishing chronic infection [3,6]. Chronic infection is maintained through a balance between controlling the parasite and limiting inflammatory tissue damage; disruption of immune control may lead to reactivation of latent infection and the development of toxoplasmic encephalitis, particularly in immunocompromised individuals [6,7].

Toxoplasmic encephalitis (TE) is a severe opportunistic manifestation of *T. gondii* infection that occurs predominantly following reactivation of latent infection in individuals with impaired cell-mediated immunity. It is particularly important in patients with advanced HIV infection, but may also occur in transplant recipients and individuals receiving immunosuppressive therapies. In susceptible patients, TE may cause severe neurological disease and can be life-threatening if not appropriately recognized and treated [7,8]. Preventive strategies, including appropriate chemoprophylaxis in high-risk seropositive individuals, therefore remain important for reducing the risk of TE in immunocompromised populations [9].

CD68 is a transmembrane glycoprotein expressed at high levels in mononuclear phagocytes, including monocytes, macrophages, and microglia, and is localized predominantly to the endosomal–lysosomal compartment [10,11]. Owing to this cellular localization, CD68 immunoreactivity reflects not only the number of microglial cells but also the endosomal–lysosomal and phagolysosomal activity associated with microglia/macrophages and infiltrating monocytes. In central nervous system pathologies, an increased CD68 response has been associated with phagocytic activity and the clearance of myelin degradation products in active multiple sclerosis lesions, an activation-associated microglial phenotype in Alzheimer’s disease, altered microglial/macrophage polarization and NADPH oxidase 2-associated neuroinflammation in traumatic brain injury, and the temporal evolution of inflammatory and reparative myeloid responses in intracerebral haemorrhage [12,13,14,15]. In persistent *T. gondii* infection, myeloid-derived cells, including activated microglia, have been shown to surround neuronal somata and perisomatic synaptic terminals, with this alteration accompanied by the loss of inhibitory perisomatic synapses [16]. A more recent study reported that CCR2-positive monocytes and CD68 signal were concentrated particularly in foci containing replicating tachyzoites during acute brain infection and that this local myeloid response was associated with active parasite replication [17]. However, that study did not assess quantitative changes in CD68 across sequential early and chronic stages of infection.

S100B is a calcium-binding protein concentrated primarily in astrocytes in the central nervous system; however, it is not entirely specific to astrocytes and may also be detected in cells with different glial or neuronal profiles [18,19]. S100B immunoreactivity has been reported to increase in association with reactive gliosis during brain injury and chronic inflammation, whereas elevated S100B levels in the cerebrospinal fluid have been associated with neuronal injury in central nervous system infections [18,20]. Increased S100B-RAGE signalling has been associated with glial inflammation, demyelination, and axonal/neuronal injury in multiple sclerosis lesions and experimental demyelination models, while blockade of this axis has been shown to reduce glial reactivity and tissue injury [21,22]. Although data directly addressing S100B immunoreactivity in *T. gondii* infection are limited, astrocytic TGF-β signalling has been reported to restrict neuroinflammation and neuronal injury, marked reactive glial alterations have been described in previous studies of experimental toxoplasmic encephalitis, and an A1-associated reactive astrocyte phenotype may develop in the infected brain [23,24,25,26]. In addition, *T. gondii*-infected neurons have been shown to exhibit reduced extracellular vesicle production and altered vesicle content; vesicles derived from these neurons induced a proinflammatory transcriptional response in astrocytes and reduced glutamate transporter 1 (GLT-1) protein levels [27]. These findings suggest that S100B should not be regarded as a direct marker for astrocyte enumeration but rather as a marker that contributes to the assessment of astroglial reactivity and the accompanying neuroinflammatory tissue alterations in toxoplasmic encephalitis.

Forkhead box P3 (Foxp3) is a key transcription factor for the differentiation of regulatory T (Treg) cells, the maintenance of their cellular identity, and the persistence of their suppressive programme [28]. However, the distribution and functional effects of Foxp3-positive cells in neuroinflammation may vary according to the nature of the disease and the inflammatory microenvironment. Foxp3-positive Treg cells have been shown to be limited in active multiple sclerosis lesions; transient reduction in Treg-mediated systemic immunosuppression in an Alzheimer’s disease model facilitated immune-cell entry into the brain and amyloid clearance; and functionally impaired Treg cells obtained from patients with Parkinson’s disease regained their suppressive properties after *ex vivo* expansion [29,30,31]. In addition, a Foxp3-positive macrophage subset distinct from conventional Treg cells has been reported to contribute to the resolution of inflammation and limitation of tissue injury through increased efferocytic capacity in acute ischaemic brain injury [32]. This finding indicates that Foxp3 positivity should not be interpreted directly as indicating Treg cells without consideration of cellular morphology and anatomical localization. In chronic *T. gondii* infection, Foxp3-positive Treg cells have been shown to accumulate particularly in the meningeal compartment, rather than being distributed widely throughout the brain parenchyma, and to establish prolonged contacts with CD11c-positive cells at this site [33]. In the chronically infected brain, ICOS signalling has been reported to contribute to the maintenance of Treg frequency and phenotypic stability while limiting the effector T-cell response; the absence of ICOS or disruption of ICOS-mediated PI3K signalling was accompanied by reduced Treg frequency, effector T-cell expansion, inadequate parasite-specific IgG production, and an increased brain cyst burden [34,35]. The loss of T-bet specifically in Treg cells during acute oral *T. gondii* infection also caused excessive Th1-mediated inflammation and mortality without a marked increase in parasite burden, demonstrating the importance of the T-bet-dependent Treg response in limiting infection-induced immunopathology [36]. These data indicate that assessing the Foxp3-positive cellular response in brain tissue at different stages of infection and within different anatomical compartments is important for elucidating the organization of local immunoregulatory components in toxoplasmic encephalitis.

IL-5 is a pleiotropic cytokine that supports the proliferation and maturation of eosinophil precursors and enhances the activation and survival of mature eosinophils; particularly in mice, it also contributes to the maintenance of B-1 cells and the differentiation of activated B cells into antibody-secreting cells [37,38]. However, the effect of IL-5 in *T. gondii* infection appears to vary according to the experimental model. Following intraperitoneal infection with ME49 tissue cysts, IL-5 transcripts were increased in the brain and spleen, whereas IL-5 deficiency was associated with higher mortality, an increased brain cyst burden, and increased tachyzoite-specific SAG2 transcripts during the chronic phase [39]. These findings suggest that IL-5 may be an immune component contributing to the control of chronic infection in the intraperitoneal ME49 model. By contrast, in acute oral infection with the RRA strain, IL-5 deficiency reduced mortality, alleviated necrotic changes and villus shortening in the small intestine, and increased systemic IL-12 and IFN-γ responses [40]. These opposing findings indicate that IL-5 cannot be uniformly classified as protective or pathogenic in *T. gondii* infection; its biological effect is shaped by the stage of infection, parasite strain, inoculation route, and affected anatomical compartment. Therefore, assessing IL-5 immunoreactivity in brain tissue from the early phase through the prolonged chronic phase in an intraperitoneal ME49 model is important for defining the temporal course of the IL-5-associated neuroimmune response in toxoplasmic encephalitis.

The aim of this study was to evaluate concurrently the temporal changes in the CD68-associated myeloid/phagolysosomal response, S100B-associated astroglial reactivity, Foxp3-associated tissue immunoreactivity, and the IL-5-associated cytokine response from the early neuroinflammatory period through the prolonged chronic phase of experimental toxoplasmic encephalitis. To our knowledge, the immunoreactivities of these four markers have not previously been evaluated together with histopathological changes within the same experimental model and at sequential stages of infection. This integrated approach was designed to define the neuroimmunopathological remodelling that extends from early myeloid/phagolysosomal activation to chronic astroglial and immunoregulatory alterations in experimental toxoplasmic encephalitis. Accordingly, the present study focuses on the temporal neuroimmune changes associated with primary and persistent cerebral *T. gondii* infection and was not designed to reproduce immunosuppression-associated reactivation of latent toxoplasmosis.

## 2. Results

### 2.1. Confirmation of Infection and General Observations

No tissue cysts were detected in the control group. Cerebral *T. gondii* infection was morphologically confirmed in the infected groups by identification of round-to-oval, bradyzoite-containing tissue cysts (Figure 1) in squash preparations and/or histological brain sections. Tissue cysts were identified across the investigated infection periods. At day 10 post-infection, cysts were relatively sparse and focally distributed in the brain tissue. At day 30, tissue cysts were more readily observed and were associated with more prominent multifocal inflammatory and glial reactions. At day 60, tissue cysts remained detectable within the brain parenchyma and were accompanied by persistent gliotic and inflammatory changes. The squash-smear examination was used as a qualitative method to confirm cerebral infection rather than for quantitative assessment of parasite burden.

Throughout the experimental period, no severe clinical signs compatible with acute toxoplasmosis, including marked lethargy, severe piloerection, pronounced hunched posture, or pre-euthanasia mortality, were observed. Neuropathological changes showed a clear temporal progression: alterations were generally mild and focal at day 10, became more prominent and multifocally distributed by day 30, and persisted at day 60 with established inflammatory, gliotic, and neuronal degenerative changes.

### 2.2. Histopathological Findings

H&E-stained brain sections from the infected groups showed multifocal inflammatory, gliotic, and neurodegenerative changes accompanied by *Toxoplasma gondii* tissue cysts. Perivascular mononuclear cell cuffing, focal glial proliferation, and glial nodule formation were observed in the brain parenchyma, together with meningeal and submeningeal mononuclear cell infiltration. Sharply delineated, round or oval, morphologically intact *T. gondii* tissue cysts were observed in the parenchyma (Figure 2A,B). Increased numbers of glial cells and a mononuclear cellular reaction were noted around the cysts. Perineuronal clearing and degenerative changes characterized by neuronal shrinkage, cytoplasmic eosinophilia, and nuclear pyknosis were observed (Figure 2B). Marked focal or multifocal gliosis, glial nodule formation, vascular hyperaemia/congestion, neuropil vacuolation, and focal neuronal necrosis were identified in other parenchymal areas (Figure 2C). Mononuclear cell infiltration in the pial and subpial regions was accompanied by superficial parenchymal gliosis, neuropil vacuolation, and neuronal degeneration. These findings were consistent with non-suppurative meningoencephalitis (Figure 2A,D).

At day 10 post-infection, the histopathological changes were predominantly mild, occurred in small foci, and had a limited distribution. The principal findings during this period were vascular hyperaemia, mild perivascular mononuclear cell cuffing, focal meningeal mononuclear cell infiltration, incipient glial proliferation, and occasional neuronal degeneration. Tissue cysts were few and focally distributed; marked glial nodule formation, widespread gliosis, and large necrotic foci were not prominent. The findings were considered consistent with the mild and focal inflammatory, vascular, and gliotic response of the early stage of infection.

At day 30 post-infection, the severity and parenchymal distribution of the lesions had increased, and the histopathological appearance had acquired the distinct characteristics of the early chronic phase. Perivascular mononuclear cell cuffing, non-suppurative meningeal inflammation, multifocal gliosis, glial proliferation, and glial nodule formation became more pronounced. Neuronal degeneration, neuropil vacuolation, and focal necrotic changes were more frequent, while the parenchymal distribution and visibility of tissue cysts increased. A glial and mononuclear cellular reaction was noted around the cysts. This appearance was consistent with cyst-associated toxoplasmic meningoencephalitis of moderate severity and multifocal distribution.

At day 60 post-infection, persistent perivascular and meningeal mononuclear inflammation, multifocal and, in places, widespread gliosis, glial nodules, neuropil vacuolation, and ongoing neuronal degenerative and necrotic changes were observed. *T. gondii* tissue cysts persisted in the parenchyma, with a marked gliotic and inflammatory reaction in the surrounding tissue. The finding that the cumulative gliotic response and neuronal injury reached their greatest severity in the setting of persistent inflammation was considered consistent with established late chronic toxoplasmic meningoencephalitis.

### 2.3. Immunoperoxidase Findings

Immunoperoxidase examination revealed distinct temporal patterns of CD68, S100B, Foxp3, and IL-5 immunoreactivity following *T. gondii* infection. CD68 immunoreactivity was significantly increased as early as day 10 and increased further by day 30, remaining at a similarly high level at day 60. In contrast, S100B, Foxp3, and IL-5 immunoreactivities at day 10 did not differ significantly from controls but increased markedly by day 30. S100B and Foxp3 remained at comparable high levels between days 30 and 60, whereas IL-5 showed a further significant increase at day 60. Thus, the four markers exhibited temporally distinct response patterns, with early CD68 activation followed by prominent chronic S100B-, Foxp3-, and IL-5-associated responses. Detailed quantitative values and pairwise statistical comparisons are presented in Table 1.

### 2.4. CD68 Immunoreactivity

In the healthy control group, CD68 immunoreactivity was very weak and was confined to a few scattered small cells with glial/myeloid morphology in the neuroparenchyma (Figure 3A). In the infected groups, pronounced granular cytoplasmic staining was observed in small and medium-sized round, oval, or irregularly shaped myeloid cells. In addition, some cells regarded as neurons on the basis of their large polygonal or pyramidal morphology showed diffuse or fine-to-coarse granular perikaryal CD68 immunoreactivity that spared the nucleus. It was particularly noteworthy that, within the same microscopic field, some cells with similar neuronal morphology showed strong perikaryal immunoreactivity, whereas neighbouring cells remained completely negative. This heterogeneous distribution of positive and negative neuronal cells within the same field was considered an important morphological finding, indicating that CD68 immunoreactivity formed a selective, cell-associated staining pattern distinct from a diffuse parenchymal background reaction (Figure 3B–F).

CD68-positive cells were markedly increased at day 10 post-infection compared with the control group and were distributed throughout the neuropil either individually or in small multifocal aggregates. At day 30, the reaction became more intense and widespread; hypertrophic or amoeboid myeloid cells with strong granular staining and large cells with a perikaryal reaction were more prominent. At day 60, the widespread and multifocal distribution of CD68-positive cells persisted, and the reaction largely maintained the high level observed at day 30. Thus, the strongest CD68 reaction was observed in the day 30 and day 60 groups, whereas the reaction in the day 10 group was greater than that in the control group but lower than those in the chronic groups.

Quantitative image analysis showed that the percentages of CD68-immunopositive area in the control, Tg day 10, Tg day 30, and Tg day 60 groups were 1.864 ± 0.375%, 6.020 ± 0.406%, 8.337 ± 0.508%, and 8.063 ± 0.426%, respectively (mean ± SD; *n* = 6). The overall difference among the groups was significant (one-way ANOVA, F(3,20) = 288.11; *p* < 0.001). CD68 immunoreactivity was higher in all infected groups than in the control group (*p* < 0.001 for all comparisons). Values in the day 30 and day 60 groups were also significantly higher than those in the day 10 group (*p* < 0.001 for both comparisons), whereas no significant difference was found between days 30 and 60 (*p* = 0.695) (Figure 4A). CD68 therefore showed an early rise followed by sustained high immunoreactivity throughout the chronic phase.

### 2.5. S100B Immunoreactivity

In the control group, S100B immunoreactivity was weak and consisted of limited fibrillary staining associated with a few scattered small cells with glial morphology in the neuroparenchyma (Figure 5A). In the infected groups, marked fibrillary immunoreactivity was observed in glial cell bodies and processes in the superficial–subpial parenchyma and around small vessels (Figure 5B). Strong cytoplasmic and focal nuclear reactions were detected in the choroid plexus epithelium; staining was widespread along the papillary surface but varied in intensity among cells (Figure 5C). The concentration of S100B-positive glial cell bodies and fine fibrillary processes around morphologically intact, immunonegative *T. gondii* tissue cysts formed a distinct pericystic glial reaction (Figure 5D,E). The most striking focal alteration was observed around a dense aggregate of mononuclear cells. While most cells at the centre of the inflammatory focus were negative or weakly stained, the reaction was markedly stronger in glial cells and their processes at the periphery of the focus. Thus, S100B immunoreactivity formed a distinct perilesional glial pattern concentrated particularly at the margins of inflammatory lesions (Figure 5F).

When the groups were compared, S100B immunoreactivity showed a slight increase in the day 10 group relative to the control group, although the difference was not significant (4.226 ± 0.496% and 3.555 ± 0.452%, respectively; *p* = 0.082). At day 30, the intensity and extent of staining increased markedly in the superficial–subpial and perivascular regions, in small glial cells within the neuroparenchyma, and around inflammatory foci (7.771 ± 0.506%). At day 60, the same cellular and regional distribution was maintained, while S100B immunoreactivity reached its numerically highest level (8.077 ± 0.349%). The day 30 and day 60 groups showed significantly greater immunoreactivity than both the control and day 10 groups (*p* < 0.001 for all comparisons); however, there was no significant difference between days 30 and 60 (*p* = 0.656). The overall difference among the groups was significant (one-way ANOVA, F(3,20) = 159.72; *p* < 0.001) (Figure 4B). In contrast to CD68, the S100B response was minimal early in infection but became prominent by day 30 and persisted through day 60.

### 2.6. Foxp3 Immunoreactivity

Foxp3 immunostaining revealed nuclear reactivity in small mononuclear cells, together with heterogeneous nuclear, perinuclear, and cytoplasmic staining in other cellular profiles. In the control group, immunoreactivity was absent or confined to very rare cells; no perivascular or meningeal clustering of positive cells was observed (Figure 6A). In the infected groups, linear and perinuclear reactions were observed together with nuclear-positive small mononuclear cells along ventricular and ependymal lining surfaces (Figure 6B). Prominent immunoreactivity was observed in cellular profiles associated with the choroid plexus and ventricular surface; the identity of all stained cells in these regions could not be established by morphology alone (Figure 6C). A granular or homogeneous perikaryal reaction was detected in some large cells with neuronal morphology in the neuroparenchyma (Figure 6D), while nuclear, perinuclear, and cytoplasmic staining was observed in cells with glial and neuronal morphology in other areas (Figure 6E). At the vessel–parenchyma interface, immunoreactivity was observed in vessel-associated cellular profiles and along the vascular wall, with additional staining in surrounding parenchymal cells. The identity and precise subcellular localization of the signal in all arrowhead-marked profiles could not be established unequivocally from this image alone (Figure 6F). Overall, Foxp3-positive small mononuclear cells were concentrated primarily in perivascular, meningeal, and ventricular inflammatory regions, while heterogeneous reactions in neuronal and glial cells accompanied this cellular response.

When the groups were compared, Foxp3 immunoreactivity in the day 10 group was weak and limited and did not show a significant increase relative to the control group (1.292 ± 0.204% and 0.895 ± 0.339%, respectively; *p* = 0.176). At day 30, the density of nuclear-positive mononuclear cells, particularly in the perivascular, meningeal, and ventricular regions, and the intensity of parenchymal staining increased markedly (4.794 ± 0.455%). At day 60, the same regional distribution was maintained, while immunoreactivity reached its numerically highest level (5.053 ± 0.227%). The day 30 and day 60 groups showed significantly greater immunoreactivity than both the control and day 10 groups (*p* < 0.001 for all comparisons); by contrast, no significant difference was found between the day 30 and day 60 groups (*p* = 0.517). The overall difference among the groups was also significant (one-way ANOVA, F(3,20) = 285.40; *p* < 0.001) (Figure 4C). Following a limited early response, Foxp3 immunoreactivity increased markedly at day 30 and remained elevated at day 60, with no statistically significant difference between these two time points.

### 2.7. IL-5 Immunoreactivity

In the control group, IL-5 immunoreactivity was virtually absent and was confined to rare, faint cellular reactions (Figure 7A). In the infected groups, cytoplasmic and perinuclear staining was observed in ependymal cells lining the ventricular space and in the adjacent periventricular parenchyma (Figure 7B). In the neuroparenchyma, granular cytoplasmic reactions in small cells with glial/mononuclear morphology were accompanied by multifocal immunoreactivity in the neuropil (Figure 7C). In large polygonal or pyramidal cells with neuronal morphology, the IL-5 reaction appeared as granular or homogeneous perikaryal staining that spared the nucleus (Figure 7D). In other parenchymal regions, a heterogeneous distribution was observed, with concurrent staining of large cells with neuronal morphology and small glial cells (Figure 7E). Mild-to-moderate reactions were observed in the hippocampal granule cell layer and the surrounding neuropil, whereas more pronounced cytoplasmic staining was detected in small cells with glial morphology (Figure 7F). Thus, IL-5 immunoreactivity showed a broad distribution encompassing the ependymal–periventricular regions, neuroparenchyma, hippocampal cell layers, large cells with neuronal morphology, and small glial cells.

When the groups were compared, IL-5 immunoreactivity in the day 10 group was weak and focal and did not show a significant increase relative to the control group (1.632 ± 0.143% and 1.223 ± 0.443%, respectively; *p* = 0.241). At day 30, the intensity and extent of staining increased markedly, particularly in the periventricular regions, small glial cells, the perikarya of large cells with neuronal morphology, and the surrounding neuropil (4.112 ± 0.375%). At day 60, the same cellular and regional distribution was maintained, but the reaction extended across broader parenchymal areas and reached its highest level (4.848 ± 0.412%). The day 30 and day 60 groups showed significantly greater immunoreactivity than both the control and day 10 groups (*p* < 0.001 for all comparisons). In addition, the immunopositive area at day 60 was 17.9% greater than that at day 30, and this increase was also significant (*p* = 0.0109). The overall difference among the groups was significant (one-way ANOVA, F(3,20) = 146.53; *p* < 0.001) (Figure 4D). The IL-5 response remained limited during the early phase, became pronounced at day 30, and, unlike the chronic plateau pattern observed for the other markers, continued to increase significantly through day 60.

## 3. Discussion

Tissue cysts formed through the differentiation of proliferating tachyzoites into bradyzoites during acute infection play a critical role in maintaining parasite persistence during the chronic phase, particularly in the central nervous system [3,4,6,41]. This biological continuum provides the basis for evaluating the glial, myeloid, and immunoregulatory alterations detected in our study at 10, 30, and 60 days post-infection during the transition from the acute to the chronic phase. Within this framework, CD68 was the only marker to show a marked increase in immunoreactivity at day 10, demonstrating that myeloid/phagolysosomal activation was the predominant feature of the early neuroinflammatory period. The emergence of S100B, Foxp3, and IL-5 responses at day 30, together with multifocal gliosis, perivascular–meningeal inflammation, and neuronal injury, demonstrated the substantial involvement of glial and immunoregulatory components in the neuropathological profile during progression to chronic infection. At day 60, CD68, S100B, and Foxp3 immunoreactivities maintained the high levels observed at day 30, whereas IL-5 continued to increase significantly, constituting the most striking temporal finding of the study. This differentiated response pattern indicates that late chronic toxoplasmic encephalitis is not a quiescent terminal stage but a dynamic neuroimmunopathological period in which the cytokine microenvironment continues to be remodelled against a background of established myeloid, glial, and regulatory responses.

CD68 is a transmembrane glycoprotein expressed at high levels in mononuclear phagocytes, including monocytes, macrophages, and microglia, and is localized mainly to the endosomal–lysosomal compartment [10,11]. Because of this cellular localization, an increase in tissue CD68 should not be regarded as a direct count of microglia, but as a phagolysosomal response that reflects both the accumulation of CD68-positive myeloid cells and an increased endosomal–lysosomal burden. Because CD68 is present in both resident microglia and infiltrating monocytes/macrophages, it is appropriate to describe the immunoreactivity as a more inclusive and scientifically accurate ‘myeloid/phagolysosomal response’ rather than as ‘microglial’ immunoreactivity [11]. This interpretation is also supported by a systematic review of postmortem brain samples from patients with Alzheimer’s disease. That review reported that increased CD68 reflects an activation-associated endosomal–lysosomal phenotype rather than a simple increase in the total number of microglia [13]. Similarly, microglia and macrophages in early active multiple sclerosis lesions have been shown to express molecules associated with phagocytosis, oxidative injury, antigen presentation, and T-cell costimulation, whereas in chronic active lesions, activated myeloid cells persist for prolonged periods at the lesion margin and accompany ongoing tissue injury [12,42]. The nature of this myeloid response in toxoplasmic encephalitis varies according to the stage of infection. Orchanian et al. [17] showed that CCR2-positive monocytes, IBA1-positive myeloid cells, and CD68 signal were concentrated particularly in foci containing replicating tachyzoites during acute brain infection; this local response developed together with increases in CCL2 and NF-κB, and monocyte migration depended on live, replication-competent parasites. In chronic ME49 infection, blood-derived macrophages have been reported to exhibit a stronger NF-κB and inflammatory transcriptional programme than microglia, whereas gasdermin-D-dependent release of IL-1α from microglia contributed to brain inflammation, myeloid-cell infiltration, and parasite control [43]. The neuropathological effect of persistent infection is not limited to inflammatory foci; perisomatic inhibitory synapses have also been shown to decrease in the hippocampus and neocortex, while myeloid-derived cells, including activated microglia, surround neuronal somata and perisomatic synaptic terminals [16]. Our previous studies using a toxoplasmic encephalitis model showed that the NO/NOS response, GFAP and neurofilament expression, oxidative stress, apoptotic mechanisms, and GMF-β-associated reactive glial changes followed different temporal patterns throughout infection [24,44,45,46]. The marked early increase in SAP, persistence of NF-κB, caspase-1, and TNF-α responses at different stages of infection, and later emergence of increases in IL-1β and C3 further support that toxoplasmic neuroinflammation is neither confined to a single time point nor synchronous [47]. Within this literature, one of the most notable findings of our study was that CD68 immunoreactivity became evident during the early phase of infection, increased further at the onset of the chronic phase, and was maintained at a similarly high level during the late chronic phase. The presence of hypertrophic and amoeboid cellular profiles together with granular cytoplasmic immunoreactivity in small and medium-sized myeloid cells supports that the quantitative increase developed alongside morphological alterations consistent with activation. This temporal and morphological pattern indicates that the CD68-positive myeloid/phagolysosomal response is not a secondary event that emerges late in toxoplasmic encephalitis, but a fundamental neuroinflammatory component that becomes rapidly established during the early stage, when replicating parasites are present, and remains strongly maintained during the chronic phase, when the parasite persists as tissue cysts. When considered together with the neuron–myeloid cell contact and inhibitory synaptic remodelling described by Carrillo et al. [16], the sustained CD68 response strongly indicates the persistence of the myeloid microenvironment that shapes chronic toxoplasmic neuropathology. Although CD68 is widely used as a marker associated with the monocyte/macrophage lineage and microglia, its cellular expression is not absolutely restricted to the myeloid compartment; variable levels of CD68 mRNA and protein have also been demonstrated in fibroblasts, endothelial cells, lymphocytes, and tumour cells of different origins [10,48]. In this context, the granular perikaryal reaction that spared the nucleus and was detected in large polygonal or pyramidal cells regarded as neurons on the basis of their morphology was particularly noteworthy. The coexistence of positive and negative large cells within the same tissue field indicates that this reaction formed a heterogeneous, cell-associated pattern distinct from diffuse parenchymal background staining. Our findings provide a novel morphological observation of CD68 immunoreactivity in cells with neuronal morphology; however, confirmation of their cellular identity will require cell-type-specific colocalization studies.

S100B is a calcium-binding protein concentrated primarily in astrocytes in the central nervous system and, at high extracellular levels, is associated with RAGE (receptor for advanced glycation end products)-mediated glial inflammatory responses [19,21]. The association of elevated cerebrospinal fluid S100B levels with neuronal injury in central nervous system infections, together with the prominence of S100B in reactive astrocytes and RAGE in activated microglia/macrophages in active and chronic active multiple sclerosis lesions, supports this interpretation [20,21]. In addition, blockade of the S100B-RAGE interaction reduced glial reactivity, preserved axonal and synaptic integrity, and promoted remyelination in experimental demyelination [22]. Taken together with these data, the S100B increase in our study that became pronounced at day 30 and was maintained through day 60 suggests that the S100B-associated astroglial response is not a transient accompaniment to acute inflammation but a persistent neuropathological component established within the chronic toxoplasmic encephalitis microenvironment. Considered together with the earlier increase in CD68, early myeloid/phagocytic activation therefore preceded the more prominent astroglial response observed during chronic infection.

Cekanaviciute et al. [23] demonstrated that astroglial TGF-β signalling was activated during infection and that suppression of this signalling in astrocytes increased early immune-cell infiltration and the astroglial NF-κB/CCL5 response, thereby exacerbating neuronal injury without altering the parasite burden. We previously showed that GMF-β expression increased at day 10 and became more pronounced at day 30 in experimental toxoplasmic encephalitis; this increase was associated particularly with reactive glial cells, foci of gliosis, and microglia/macrophages, and GMF-β may promote glial inflammation and link neuropathological processes associated with IL-33, NO, and TNF-α [46]. Still et al. [49] further showed that IL-33 expressed by oligodendrocytes and astrocytes during chronic infection was released into the cerebrospinal fluid and that the astrocytic ST2-mediated IL-33 response was required for adequate T-cell proliferation, IFN-γ production, and parasite control. In the present study, S100B immunoreactivity showed a modest numerical upward trend relative to the control at day 10, but this change did not reach statistical significance. In contrast to the marked increase in CD68 observed during the same period, this finding suggests that the S100B-associated astroglial response was not yet fully established during early toxoplasmic encephalitis. The marked increase in S100B at days 30 and 60 and the absence of a significant difference between these two periods indicate that the response reached a high level at day 30 and persisted through day 60. This temporal pattern is consistent with the glial activation- and neuronal injury-associated changes reported in previous studies of toxoplasmic encephalitis [24,45] and with the increase in GMF-β in reactive glial cells that began at day 10 and became more pronounced at day 30 [46]. Given that S100B is an important indicator of astroglial reactivity and tissue stress in neuropathological processes, S100B remained relatively low early in infection but increased markedly by day 30 and remained elevated at day 60. The marked increase in CD68 expression at day 10, in contrast to the later emergence of the S100B response, suggests that glial responses do not develop synchronously in toxoplasmic encephalitis; early phagolysosomal myeloid activation is followed by astroglial remodelling that becomes established and persistent during the chronic phase. Accordingly, the sustained increase in S100B at days 30 and 60 may be regarded as a strong indicator of a persistent astroglial activation phenotype maintained throughout chronic infection rather than as a transient indicator of inflammation.

Foxp3 is a key transcription factor for the development of regulatory T (Treg) cells, the maintenance of their cellular identity, and their suppressive programme [28]. The nature of the Foxp3 response in *T. gondii* infection may vary according to the stage of infection: acute and strong Th1 conditions can lead to a reduction in Treg-cell numbers and phenotypic alteration, whereas T-bet expression in Treg cells has been shown to be necessary for limiting excessive Th1-mediated immunopathology [36,50]. During chronic central nervous system infection, Foxp3-positive Treg cells have been reported to accumulate in the meningeal compartment and establish prolonged contacts with CD11c-positive cells [33]. In addition, the IL-10 and ICOS pathways have been shown to regulate T-cell responses in the chronically infected brain in different ways; the absence of ICOS or selective disruption of ICOS-PI3K signalling reduced Treg frequency in the brain, decreased Foxp3 and CD25 expression in Treg cells with impaired PI3K signalling, and was accompanied by effector T-cell expansion and impaired parasite control [34,35]. In our study, Foxp3-positive cells were absent or very rare in the control group, while the response remained limited at day 10 and did not differ significantly from that in the control group. By contrast, Foxp3 immunoreactivity increased markedly at day 30; nuclear staining considered specific was concentrated particularly in small, lymphocyte-like mononuclear cells located within perivascular cuffs and areas of meningeal/submeningeal inflammation. Foxp3 immunoreactivity remained elevated at day 60, with no statistically significant difference from day 30. This distribution suggests that the Foxp3-positive cellular response was organized in the vascular and meningeal compartments, which are sites of immune-cell interaction in chronic infection, rather than representing a diffuse parenchymal accumulation. Compared with the CD68-positive myeloid/phagocytic response that became evident during the early phase, the strengthening of the Foxp3 response at day 30 indicates that the regulatory cellular component became pronounced with chronicity; the similarity between days 30 and 60 indicates that this response formed a high-level plateau maintained into the late chronic phase rather than a progressive accumulation. The resulting spatial and temporal pattern therefore supports that perivascular and meningeal organization of Foxp3-positive cells is a prominent immunoregulatory component accompanying ongoing parenchymal myeloid–glial activity in chronic toxoplasmic encephalitis.

IL-5 is a pleiotropic cytokine that in humans primarily supports eosinophil development, activation, and survival and, in experimental models, regulates numerous immune processes, including the maintenance of B-1 cells and the differentiation of activated B cells into antibody-secreting cells [37,38,51]. The association of the IL-5 axis with the central inflammatory microenvironment has also been demonstrated in multiple sclerosis; the IL-5 rs2069812 CC genotype was associated with higher cerebrospinal fluid IL-5 levels and the absence of disease activity during three years of follow-up, whereas the T allele was associated with a more proinflammatory cytokine profile [52]. However, the effect of IL-5 in *T. gondii* infection appears to vary according to the experimental model. In the intraperitoneal ME49 model, IL-5 deficiency led to mortality during the chronic phase and increased brain tissue-cyst and tachyzoite burdens, while reduced IL-12 production in splenocytes stimulated with parasite antigen was associated with B-cell loss [39]. By contrast, in acute oral infection, IL-5 deficiency reduced mortality and small-intestinal pathology while increasing serum IL-12 and IFN-γ levels [40]. Taken together, these opposing findings suggest that IL-5 cannot be uniformly classified as protective or pathogenic; its effect is shaped by the stage of infection, route of infection, and affected anatomical compartment. In our study, IL-5 immunoreactivity was virtually absent or very weak in the control group, whereas the weak and focal increase observed at day 10 did not differ significantly from the control group. At day 30, IL-5 immunoreactivity became pronounced and showed a multifocal distribution in the ependymal and periventricular regions, small cells with glial morphology, the perikaryal cytoplasm of large cells with neuronal morphology, the surrounding neuropil, and regions corresponding to the hippocampal granule cell layer. At day 60, the same cellular and regional pattern persisted across broader areas and at a higher level; the IL-5 response was significantly greater not only than those in the control and day 10 groups but also than that in the day 30 group. Although the observation of staining in cells with ependymal, glial, and neuronal morphology does not prove that these structures directly produce IL-5, it indicates that the IL-5-associated tissue response in the chronically infected brain is not confined to a single cellular or anatomical compartment. In contrast to the CD68, S100B, and Foxp3 responses, which reached high but relatively stable levels during the chronic phase, the continued increase in IL-5 immunoreactivity from day 30 to day 60 was the most distinctive finding of our study. This increase, which became pronounced during the late chronic phase in the presence of persistent tissue cysts, gliosis, perivascular and meningeal inflammation, and ongoing neuronal injury, suggests that the late chronic phase of toxoplasmic encephalitis is not quiescent but represents an active and dynamic neuroimmunopathological process in which the cytokine-associated tissue microenvironment continues to be remodelled.

This study has several limitations. First, the experiments were conducted only in female Swiss albino mice; because sex was not examined as a variable, the generalizability of the findings to male animals or to differences between sexes is limited. Second, the infection model was restricted to a single parasite strain (ME49) and a single inoculation route (intraperitoneal); because the literature indicates that the protective or pathogenic effect of IL-5 may vary according to the strain and route of infection [39,40], the generalizability of the present findings to other experimental conditions, such as different strains or oral infection models, should be considered with caution.

Third, CD68, S100B, Foxp3, and IL-5 immunoreactivities were evaluated using single-marker immunoperoxidase staining; cell-type-specific dual/multiple immunofluorescence labelling or intracellular colocalization analysis was not performed. Therefore, the precise cellular source of the immunoreactivity observed in cells with neuronal morphology, particularly for IL-5 and CD68, could not be determined (microglia, infiltrating macrophages, astrocytes, neurons, or ependymal cells); the findings should be interpreted as a tissue-level immunoreactivity pattern and not as a definitive attribution of cellular origin.

Fourth, the study was descriptive and observational; because neutralization, blockade, or genetic-deficiency (knockout) approaches were not used, the relationship between the observed temporal patterns, particularly the continued increase in IL-5 during the late chronic phase, and tissue injury is associative rather than causal. Fifth, the tissue-cyst burden was not quantified or subjected to correlation analysis with immunoreactivity levels; therefore, it was not possible to distinguish whether the immune responses developed in relation to the parasite burden or to cumulative tissue injury. Finally, although the sample size of six animals per group was considered sufficient for the statistical comparisons, it may reduce the precision of effect-size estimates, particularly for comparisons approaching borderline significance. An important limitation of the present study is the absence of an experimentally immunosuppressed group. Therefore, the temporal immunohistochemical patterns identified in this model should not be directly extrapolated to reactivation-associated toxoplasmic encephalitis occurring under profound immunosuppression. Future studies incorporating pharmacological or genetic immunosuppression would be valuable for determining whether the temporal patterns observed here are maintained or altered during parasite reactivation.

These limitations should be considered when interpreting the present findings. Future studies incorporating dual labelling, functional validation, and cyst-burden correlation analyses may help clarify the mechanistic basis of the temporal immunopathological patterns observed here.

## 4. Materials and Methods

### 4.1. Ethics Statement

This study was conducted in accordance with the ethical principles governing the use of laboratory animals in scientific research. The experimental protocol was approved by the Kırıkkale University Animal Experiments Ethics Committee (Decision no. 14/15-14; date: 1 September 2014). All animal procedures and tissue collection used in the present study were performed under this approved protocol. No additional animal experimentation was conducted specifically for the preparation of the present manuscript. The study was reported in accordance with the ARRIVE 2.0 guidelines [53]. All experimental procedures were performed with due consideration for animal welfare. The animals were monitored throughout the experimental period; all necessary measures were taken to minimize pain, stress, and the number of animals used while preserving the scientific validity of the study.

### 4.2. Animals and Experimental Design

Adult female Swiss albino mice aged 12–16 weeks and weighing 22–25 g were used in this study. Female mice were used throughout the study in accordance with the original experimental protocol, thereby maintaining a consistent sex across all experimental groups. Because only female mice were included, the findings should be interpreted within this experimental context, and potential sex-related differences in the neuroimmune response to *T. gondii* infection cannot be excluded. No pharmacological or genetic immunosuppression was applied to the animals before or during the experimental period. The present experimental design was intended to characterize the temporal neuropathological and neuroimmune changes associated with primary and persistent ME49 infection rather than immunosuppression-induced reactivation of latent infection. The animals were obtained from the Saki Yenilli Laboratory Animal Breeding Laboratory, Ankara, Türkiye. The mice were housed under standard laboratory conditions and regularly monitored for survival, general clinical condition, body weight, activity, posture, coat appearance, and neurological signs until tissue sampling.

A total of 24 mice were allocated to four experimental groups (*n* = 6 per group). Six uninfected mice that received an equal volume of parasite-free sterile physiological saline served as the control group. The control animals were euthanized at the time point corresponding to day 60 post-infection so that they were age-matched to the day 60 infected group and matched to the longest follow-up period of the experiment. The remaining 18 mice were infected with the low-virulence ME49 strain of *T. gondii* and divided into three infected groups of six animals each. Infected animals were euthanized at 10, 30, and 60 days post-infection. The sample size of six animals per group was defined in the original experimental protocol with consideration of previous studies using comparable experimental toxoplasmosis models and the ethical principle of reducing animal use while maintaining sufficient biological replication for group-level comparisons. Each animal was considered an independent biological replicate and the experimental unit for statistical analysis.

For the purposes of this study, day 10 post-infection was considered the early infection/early neuroinflammatory period, day 30 the established chronic toxoplasmic encephalitis period, and day 60 the prolonged chronic/persistent neuroimmune period. The experimental infection procedure was performed as previously described [24,45].

### 4.3. Parasite Strain and Experimental Infection Model

The low-virulence cystogenic ME49 strain of *T. gondii* was selected because of its ability to establish chronic infection and form bradyzoite-containing tissue cysts in the brain. The strain was maintained in mice by serial passage of brain tissue cysts. For the preparation of the experimental inoculum, tissue cysts were obtained from the brains of donor mice previously infected intraperitoneally with the ME49 strain. Donor brain tissues were homogenized in sterile physiological saline, and the homogenates were examined by light microscopy to confirm and quantify tissue cysts. The inoculum was adjusted to contain 15–20 ME49 tissue cysts in 0.25 mL of sterile physiological saline per mouse. Animals in the infected groups received the inoculum intraperitoneally, whereas control animals received an equivalent volume of parasite-free sterile physiological saline.

The precise original source of the ME49 strain and the historical interval of serial passage could not be reliably reconstructed from the archived experimental records; therefore, no speculative information was introduced.

### 4.4. Anaesthesia, Euthanasia, and Brain Tissue Collection

At the designated time points, the animals were placed under deep anaesthesia before euthanasia. Anaesthesia was administered using ketamine at 20 mg/kg and xylazine at 5 mg/kg. After deep anaesthesia had been confirmed by the absence of the pedal withdrawal and corneal reflexes, the animals were euthanized by cervical dislocation. Following confirmation of death, the brain tissues were rapidly removed.

Immediately after the brains were removed, small pieces of fresh brain tissue were randomly selected and reserved for examination using the squash-smear technique to confirm the presence of *T. gondii* tissue cysts. The remaining brain tissues were fixed in 10% neutral-buffered formalin for 48 h for histopathological and immunohistochemical analyses. After fixation, the tissues were washed overnight under running tap water and processed using routine histological procedures.

### 4.5. Application of the Squash-Smear Technique for Rapid Detection of Brain Tissue Cysts

The squash-smear technique previously described by Dincel [46] was used for the rapid and direct morphological detection of cerebral *T. gondii* tissue cysts. Five small pieces of fresh brain tissue randomly selected from each infected animal were placed on poly-L-lysine-coated glass slides and gently compressed with a second slide to prepare thin squash smears. The preparations were fixed in 10% neutral-buffered formaldehyde for 1 min and passed through a graded alcohol series for 1 min for rehydration. Mayer’s haematoxylin was then applied to the preparations for 30 s, after which they were rinsed with distilled water. The preparations were subsequently dehydrated in a graded alcohol series for 1 min, cleared in xylene for 2 min, and coverslipped.

Unstained native squash preparations and preparations stained with Mayer’s haematoxylin were examined by light microscopy (Olympus, Tokyo, Japan). The presence of round or oval *T. gondii* tissue cysts containing bradyzoites was evaluated. This approach allowed the cysts to be visualized rapidly in fresh brain tissue before conventional routine tissue processing was completed and provided direct morphological confirmation that the experimental infection had reached the central nervous system.

### 4.6. Histopathological Examination

Formalin-fixed brain tissues were subjected to routine histological processing. The tissues were dehydrated through graded alcohols, cleared in xylene, and embedded in paraffin blocks. Serial sections 4–5 µm thick were cut from the paraffin blocks and mounted on glass slides, and sagittal brain sections were prepared from control and infected animals.

The sections were stained with haematoxylin and eosin and examined using an Olympus BX51 light microscope. Histopathological evaluation included morphological assessment of the presence, morphology, and parenchymal distribution of *T. gondii* tissue cysts, as well as non-suppurative meningoencephalitis, perivascular mononuclear cell cuffing, meningeal and submeningeal mononuclear cell infiltration, focal or multifocal gliosis, glial proliferation, glial nodule formation, neuronal degeneration and necrosis, neuropil vacuolation, vascular hyperaemia/congestion, the distribution of inflammatory foci, and parenchymal tissue injury.

### 4.7. Primary Antibodies

The immunohistochemical examinations used a rabbit polyclonal anti-CD68 antibody for CD68 (Abcam, Cambridge, UK; catalogue no. ab125212; working dilution 1:100–1:200), a recombinant rabbit monoclonal anti-S100B antibody [4V8R6] for S100B (Thermo Fisher Scientific/Invitrogen, Waltham, MA, USA; catalogue no. MA5-42683; working dilution 1:100), a rat monoclonal anti-Foxp3 antibody [FJK-16s] for Foxp3 (Thermo Fisher Scientific/eBioscience, Waltham, MA, USA; catalogue no. 14-5773-82; final concentration 5 µg/mL), and a rabbit polyclonal anti-IL-5 antibody for IL-5 (Thermo Fisher Scientific/Invitrogen, Waltham, MA, USA; catalogue no. PA5-99397; working dilution 1:100).

### 4.8. Immunoperoxidase Staining

To evaluate CD68, S100B, Foxp3, and IL-5 immunoreactivity, the indirect streptavidin–biotin immunoperoxidase method was applied to 4–5 µm-thick sections prepared from formalin-fixed, paraffin-embedded brain tissues. Sections mounted on adhesive slides were deparaffinized in three separate xylene baths for 5 min each, rehydrated through descending concentrations of alcohol, and rinsed with distilled water. Heat-induced epitope retrieval was performed by heating the sections in pH 6.0 citrate buffer for 20 min. Endogenous peroxidase activity was blocked by incubating the sections at room temperature for 7 min with 3% hydrogen peroxide prepared in absolute methanol. Between staining steps, the sections were washed three times for 5 min each in pH 7.4 phosphate-buffered saline. Blocking serum was applied to the sections for 5 min to reduce nonspecific antibody binding. The sections were then incubated with primary antibodies against CD68, S100B, Foxp3, and IL-5 in a humidified chamber at room temperature for 60 min. Biotinylated secondary antibody was applied for 15 min, followed by streptavidin–peroxidase conjugate for 15 min. The immunoreaction was visualized using 3-amino-9-ethylcarbazole as the chromogen, and colour development was monitored for 5–10 min. The sections were counterstained with Mayer’s haematoxylin for 1–2 min and coverslipped using an aqueous mounting medium. In each staining run, negative-control sections in which the primary antibody was omitted but all other steps were performed identically were evaluated together with control sections known to be positive for the corresponding marker. Immunostained sections were examined by light microscopy, and the fields used for histomorphometric evaluation were imaged under a 20× objective.

### 4.9. Histomorphometric Image Analysis

The tissue area occupied by immunopositive staining was measured using a computer-assisted image-analysis system comprising a Leica DFC420 CCD camera, Leica DM4000 B microscope, and Leica QWin Plus v3 software. Histomorphometric analysis was performed according to a computer-assisted image-analysis approach previously used in experimental toxoplasmic encephalitis [24,44]. For each marker, all sections were evaluated using identical microscope, camera, illumination, and software-analysis settings. For qualitative assessment of immunohistochemical staining, whole sagittal brain sections were examined, and meningeal, submeningeal, perivascular, ependymal–periventricular, choroid plexus, hippocampal, pericystic, and perilesional staining patterns were recorded morphologically. Quantitative histomorphometric analysis was performed only within the brain parenchyma. For each animal, five randomly selected, non-overlapping microscopic parenchymal fields were imaged under a 20× objective from comparable sagittal brain sections with adequate tissue integrity. Fields were not selected according to the intensity of immunostaining. The meninges, choroid plexus, ventricular space, large vascular lumina, and section margins were excluded from the quantitative analysis. Fields containing tissue folds, tears, tissue loss, chromogen deposits, or marked background staining were excluded from evaluation. Qualitatively defined meningeal, ependymal, choroid plexus, and other regional staining patterns were not included in calculations of the percentage immunopositive area in the parenchyma.

For CD68, S100B, Foxp3, and IL-5, the AEC-positive chromogenic signal morphologically consistent with the expected cellular localization of the respective marker was separated from the total haematoxylin-counterstained tissue area by colour thresholding. For Foxp3, the quantified area included nuclear, perinuclear, and cytoplasmic staining within the selected parenchymal fields, after exclusion of nonspecific background and technical artefacts. Quantification was not restricted to nuclear staining in small mononuclear cells. The resulting percentage therefore represents tissue-level anti-Foxp3 immunoreactivity rather than a selective measure of Foxp3-positive mononuclear cells. Positive areas identified by the software were verified against microscopic morphology to prevent nonspecific background staining and technical artefacts from being included in the measurements. The immunopositive area measured in each image was divided by the total evaluable tissue area in the same image and expressed as the percentage immunopositive area. The animal-level value was calculated as the mean of the five fields from each animal. All sections were evaluated independently by two investigators blinded to group and time-point information; the arithmetic mean of the values obtained by the two investigators for each animal was used as the final measurement. Microscopic fields were not treated as independent observations in the statistical analyses; each animal was considered one independent biological replicate and experimental unit.

### 4.10. Statistical Analysis

Each experimental group comprised six independent biological replicates, with the animal considered the experimental unit. For each marker, the measurements obtained from five microscopic fields in each animal were averaged, and this single animal-level value was included in the statistical analysis. This approach prevented pseudoreplication that could have resulted from treating microscopic fields from the same animal as independent observations. Continuous variables are presented as the animal-level mean ± standard deviation.

Data for CD68, S100B, Foxp3, and IL-5 were analysed independently. Before parametric analysis, residuals from the one-way analysis of variance models were assessed for marked deviations from normality using the Shapiro–Wilk test, and homogeneity of variances across groups was evaluated using the median-centred Levene/Brown–Forsythe test. Because there was no clear evidence of assumption violation, overall differences among the control, day 10 post-infection, day 30 post-infection, and day 60 post-infection groups were evaluated by a separate one-way analysis of variance for each marker. For analyses with a significant overall group effect, all prespecified pairwise group comparisons were performed using Tukey’s honestly significant difference test. Eta-squared (η^2^) and small-sample-bias-adjusted omega-squared (ω^2^) effect sizes were calculated to indicate the magnitude of the overall group effect.

A nonparametric sensitivity analysis using the Kruskal–Wallis test was also performed to assess the robustness of the parametric results. Following significant Kruskal–Wallis results, groups were compared pairwise using two-sided exact Mann–Whitney U tests, and Holm adjustment was applied to the *p* values from the six pairwise comparisons performed for each marker. The nonparametric sensitivity analyses confirmed the conclusions regarding statistical significance obtained using one-way analysis of variance and Tukey’s test. All tests were two-sided, and *p* < 0.05 was considered statistically significant. Because there were no missing data, no data imputation was performed, and no observations were excluded from the analysis. Statistical analyses were performed in Python 3.12.13 using NumPy 2.3.5 and SciPy 1.17.0.

## 5. Conclusions

This study showed that experimental toxoplasmic encephalitis is a stage-specific neuroimmunopathological process that progresses from early inflammatory alterations to persistent chronic neuroinflammation. The CD68 and S100B findings demonstrated that early myeloid/phagocytic activation is followed by astroglial reactivity and gliotic parenchymal remodelling that become pronounced during the chronic phase. The Foxp3 and IL-5 findings showed that perivascular–meningeal regulatory cellular organization and a phase-dependent cytokine response develop with chronicity. The most striking finding of our study was that IL-5 immunoreactivity continued to increase during the late chronic phase, whereas the CD68-, S100B-, and Foxp3-associated responses reached high but stable levels during the chronic stages. This temporal divergence demonstrates that late chronic toxoplasmic encephalitis is not a fixed terminal stage but a dynamic immunopathological process in which the cytokine microenvironment continues to be remodelled against a background of established myeloid, astroglial, and regulatory cellular responses. These findings raise the possibility that neuroimmune activity may persist during chronic infection and underscore the need for further investigation in clinically relevant, specifically designed models of immunosuppression and parasite reactivation to determine whether similar temporal patterns also occur during reactivation in immunocompromised hosts. By comprehensively defining the transition from the early inflammatory phase to established chronic neuroinflammation in toxoplasmic encephalitis through myeloid, astroglial, regulatory, and cytokine-associated tissue responses, this study provides a tissue-based neuropathological framework for future investigations of stage-specific disease mechanisms.

## Figures and Tables

**Figure 1:**
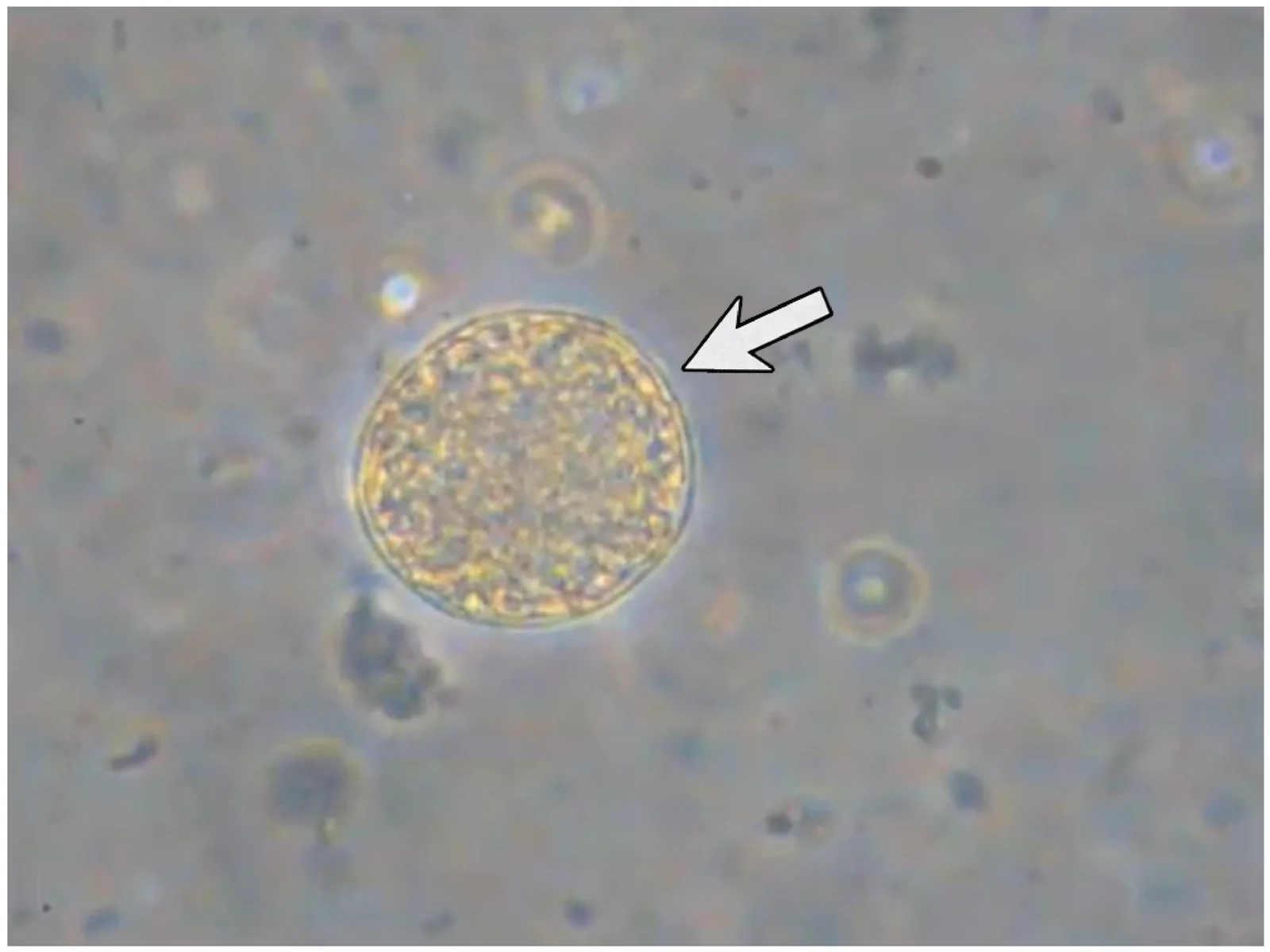
Unstained native squash preparation of fresh brain tissue obtained at day 30 post-infection from a *Toxoplasma gondii*-infected mouse showing a round tissue cyst (arrow) containing numerous bradyzoites. Light microscopy. Magnification: 400×.

**Figure 2:**
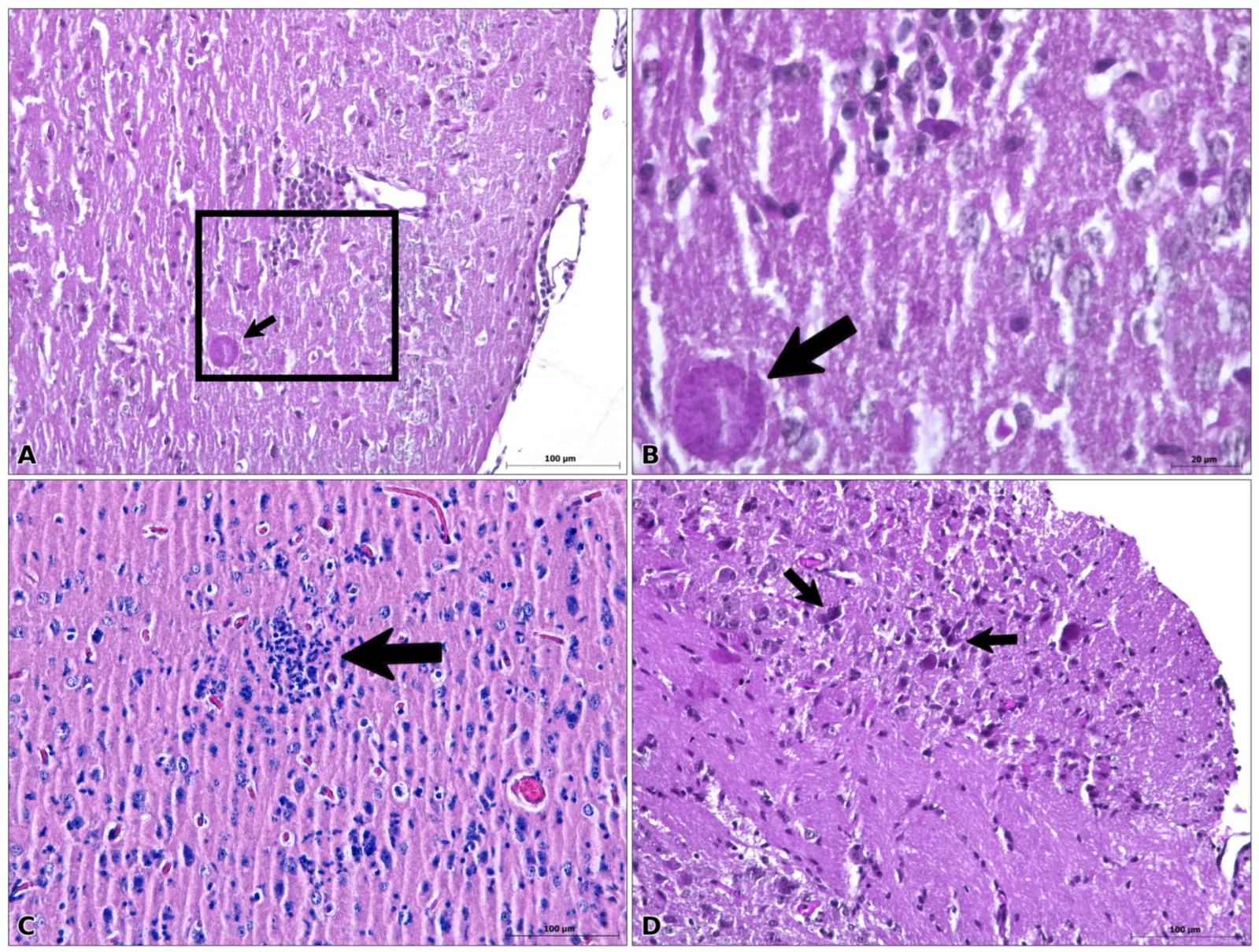
Representative histopathological changes in the brains of mice with experimental toxoplasmic encephalitis at 10, 30, and 60 days post-infection. (**A**) Day 10: a small, well-circumscribed intraparenchymal *Toxoplasma gondii* tissue cyst (arrow) accompanied by mild focal perivascular mononuclear cell cuffing and incipient glial proliferation. (**B**) Higher-magnification view of the boxed area in panel A, showing a *T. gondii* tissue cyst (arrow) and associated cellular changes. A morphologically intact tissue cyst containing bradyzoites, surrounded by a glial and mononuclear cellular reaction, with adjacent neuronal degeneration characterized by perineuronal clearing, cytoplasmic eosinophilia, and nuclear pyknosis. (**C**) Day 30: multifocal gliosis and glial nodule (arrow) formation associated with vascular hyperaemia/congestion, neuropil vacuolation, and degenerative or necrotic neuronal profiles. (**D**) Day 60: persistent pial and subpial mononuclear inflammation accompanied by superficial parenchymal gliosis, neuropil vacuolation, and neuronal degeneration (arrows), consistent with established chronic non-suppurative meningoencephalitis. H&E staining. Scale bars: (**A**,**C**,**D**) = 100 µm; (**B**) = 20 µm.

**Figure 3:**
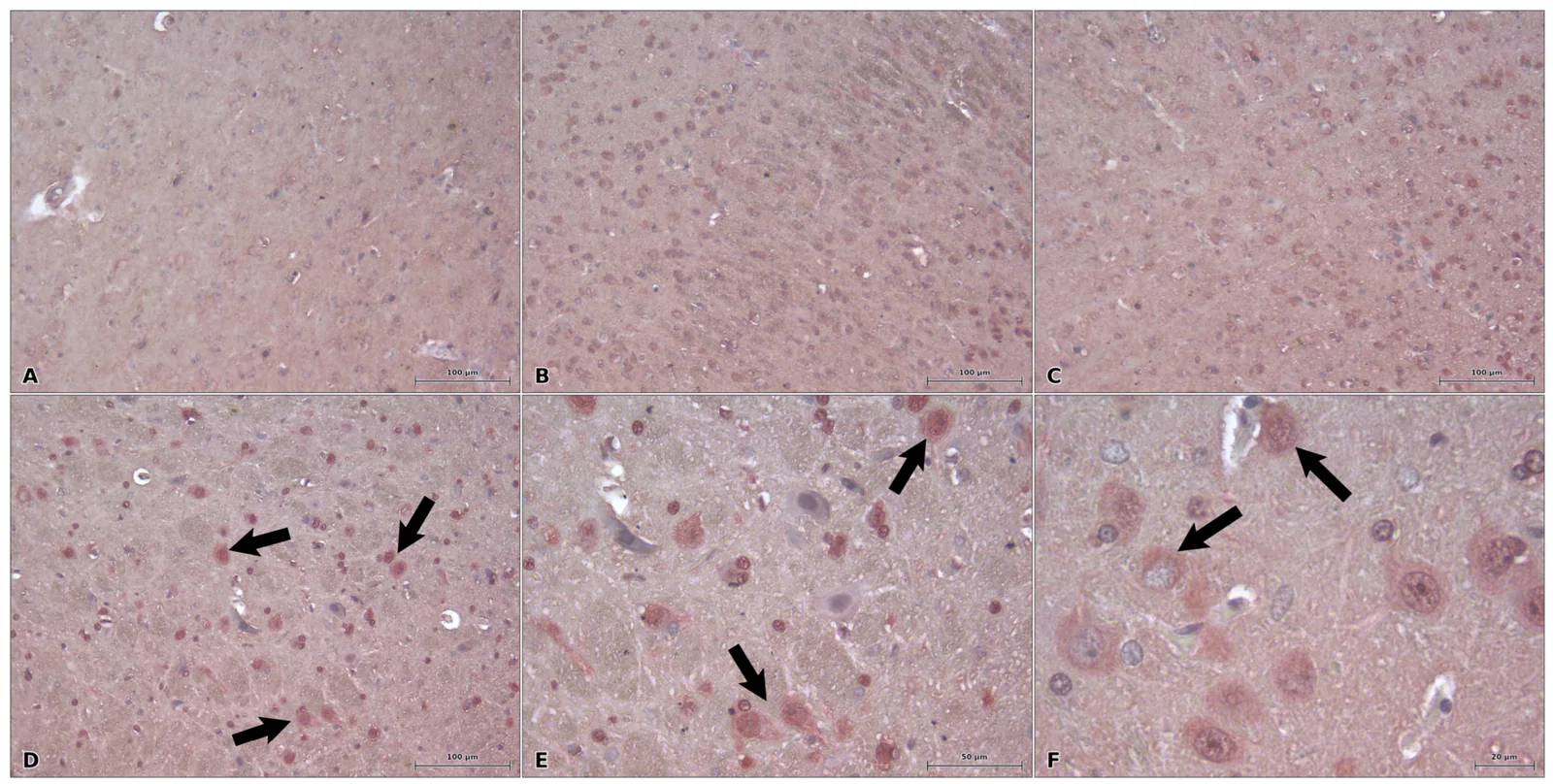
Representative temporal distribution of CD68 immunoreactivity in the brains of control and Toxoplasma gondii-infected mice. (**A**) Control: very weak immunoreactivity restricted to sparse, scattered cells with glial or myeloid morphology. (**B**) Day 10: an early increase in fine-granular cytoplasmic immunoreactivity in scattered small- to medium-sized cells with myeloid morphology. (**C**,**D**) Day 30: more intense and widespread multifocal CD68 immunoreactivity (arrows) involving numerous hypertrophic or amoeboid myeloid cells and occasional large polygonal cells with neuronal morphology. (**E**,**F**) Day 60: persistent strong immunoreactivity in myeloid cells and heterogeneous fine-to-coarse granular perikaryal staining (arrows) in large cells with neuronal morphology, predominantly sparing the nuclei. CD68 immunohistochemistry, AEC chromogen, Mayer’s haematoxylin counterstain. Scale bars: (**A**–**D**) = 100 µm; (**E**) = 50 µm; (**F**) = 20 µm.

**Figure 4:**
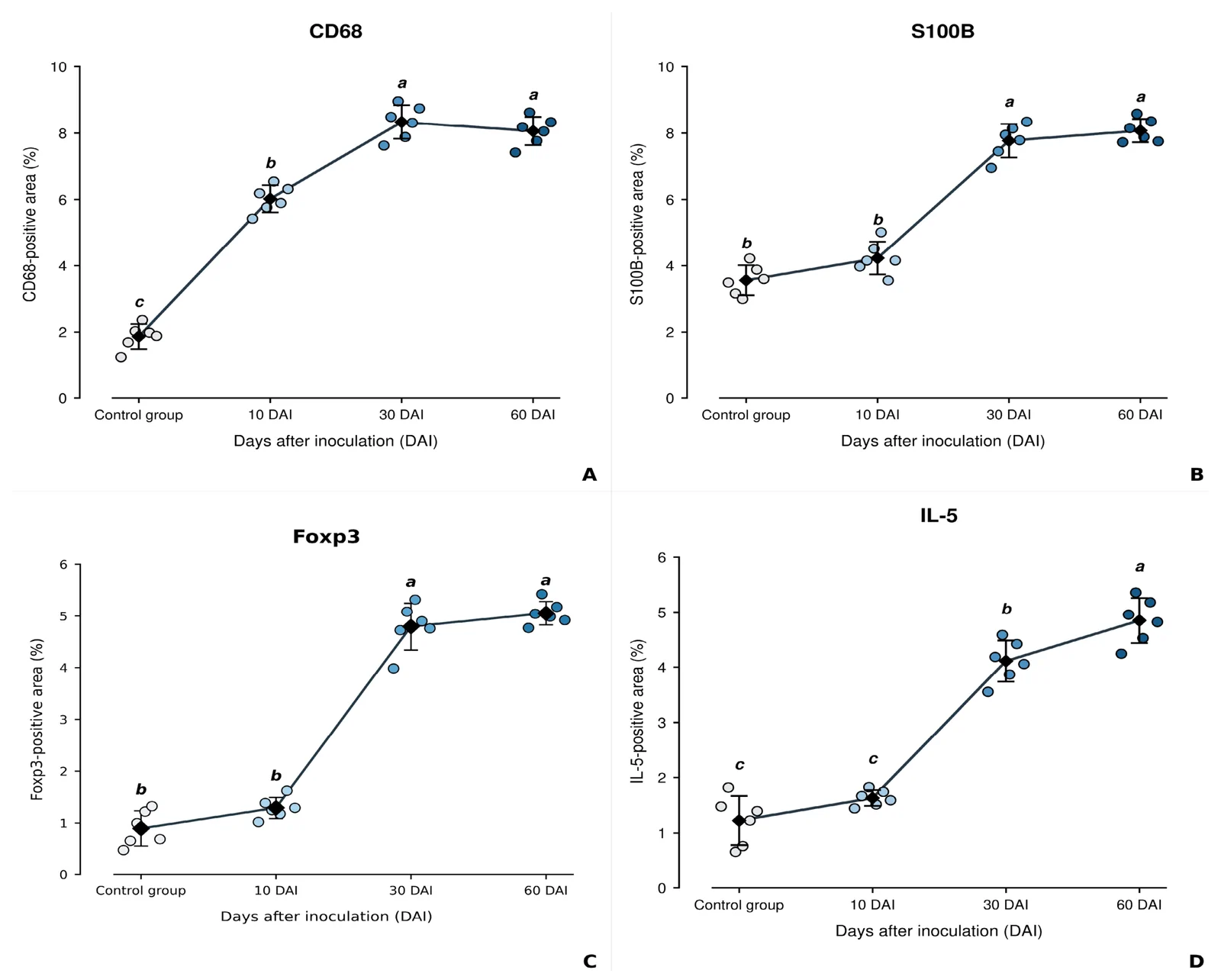
Quantification of CD68, S100B, Foxp3, and IL-5 immunoreactivity in the brain parenchyma of control and *T. gondii*-infected mice. Immunopositive area (%) for (**A**) CD68, (**B**) S100B, (**C**) Foxp3, and (**D**) IL-5 in controls and infected mice at 10, 30, and 60 days after inoculation (DAI; *n* = 6 animals per group). Circles represent individual animal values; black diamonds and error bars indicate the mean ± SD. Lines connect group means as visual guides. Within each panel, different lowercase letters indicate significant differences among groups (one-way ANOVA followed by Tukey’s HSD test, *p* < 0.05); groups sharing the same letter do not differ significantly.

**Figure 5:**
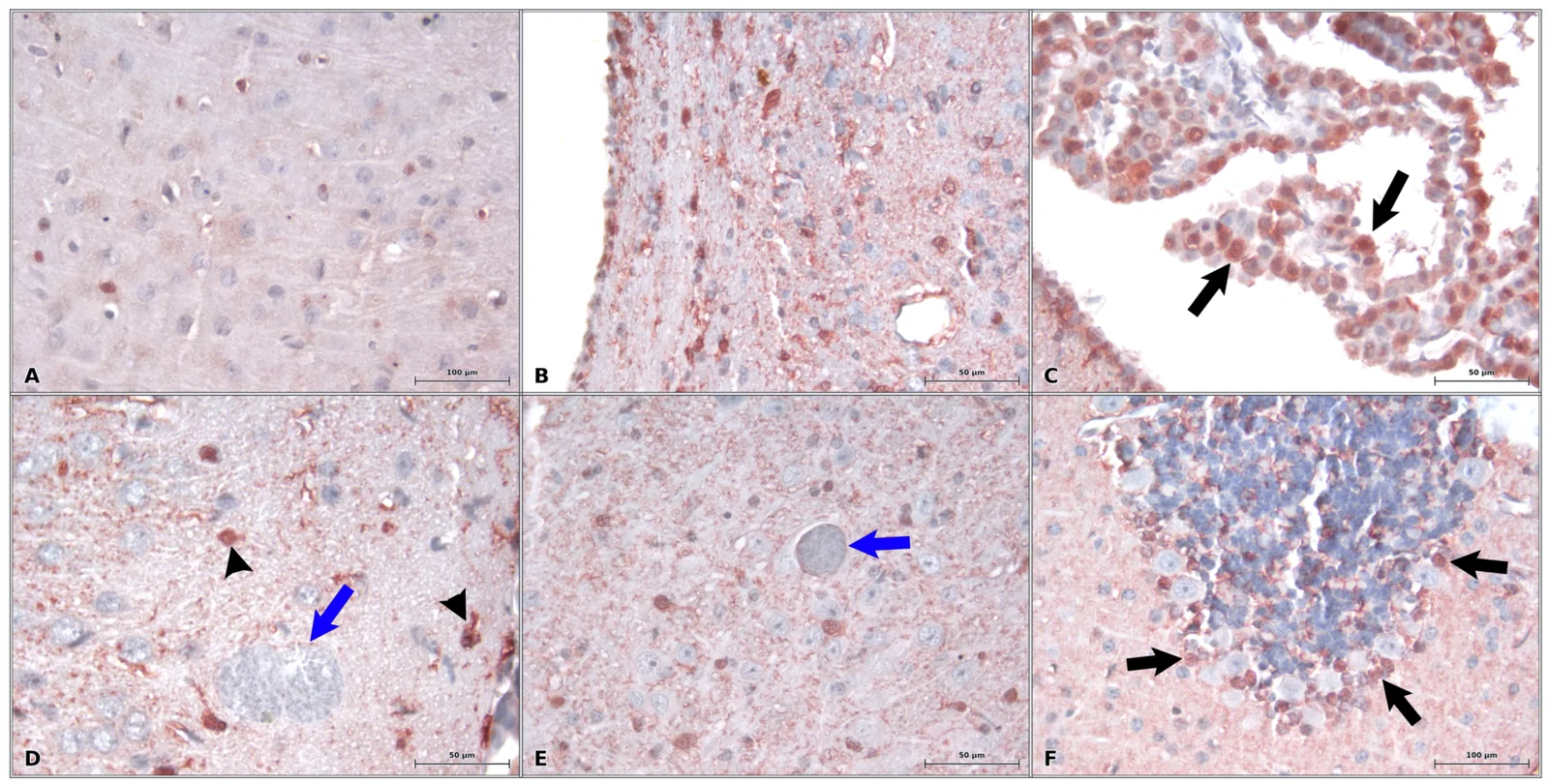
Representative temporal distribution of S100B immunoreactivity in the brains of control and Toxoplasma gondii-infected mice. (**A**) Control: weak immunoreactivity in sparse cells with glial morphology and delicate fibrillary processes within the neuropil. (**B**) Day 10: a limited early reaction characterized by S100B-positive glial cell bodies and processes in the superficial–subpial and perivascular parenchyma. (**C**,**D**) Day 30: marked S100B immunoreactivity in the choroid plexus epithelium (arrows) and in glial cell bodies (arrow heads) and processes surrounding a morphologically intact, immunonegative tissue cyst (blue arrow). (**E**,**F**) Day 60: Immunonegative tissue cyst (blue arrow), persistent and extensive pericystic and perilesional glial immunoreactivity (arrows), including enhanced staining of glial cells and fibrillary processes at the margins of dense inflammatory foci. S100B immunohistochemistry, AEC chromogen, Mayer’s haematoxylin counterstain. Scale bars: (**A**,**F**) = 100 µm; (**B–E**) = 50 µm.

**Figure 6:**
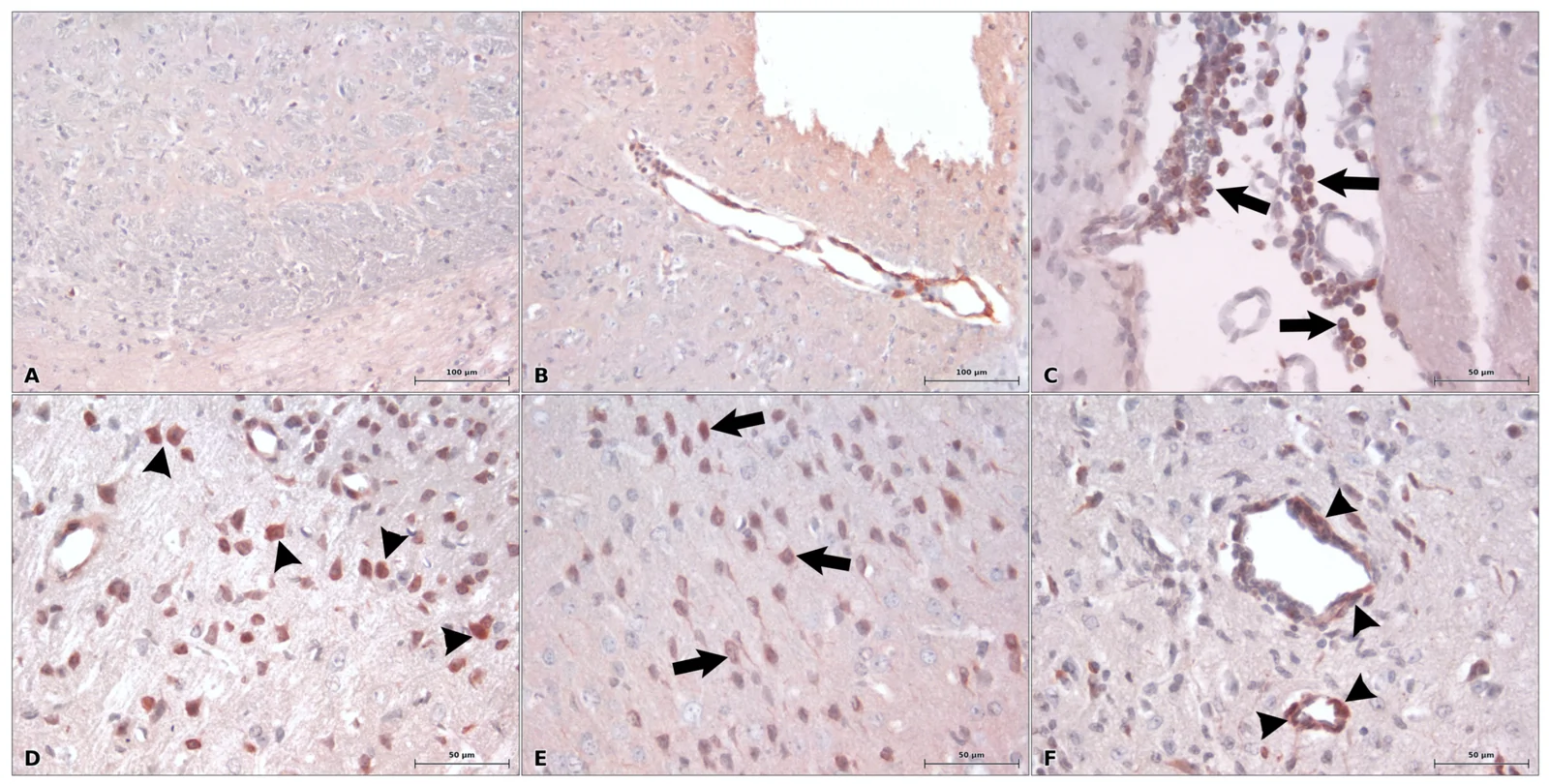
Representative temporal distribution of Foxp3 immunoreactivity in the brains of control and Toxoplasma gondii-infected mice. (**A**) Control: no convincing Foxp3 nuclear immunoreactivity or only rare, weakly stained cellular profiles. (**B**) Day 10: limited early immunoreactivity characterized by scattered nuclear-positive small mononuclear cells and linear or perinuclear staining along ventricular–ependymal and vascular interfaces. (**C**) Day 30: prominent immunoreactivity in cellular profiles associated with the choroid plexus and ventricular surface (arrows). (**D**) Day 30: heterogeneous staining in large cells with neuronal morphology (arrowheads). (**E**) Day 60: immunoreactivity in parenchymal cellular profiles with glial or neuronal morphology (arrows). (**F**) Day 60: vessel-associated immunoreactivity (arrowheads), including staining along vascular walls and in adjacent cellular profiles. Nuclear staining in small mononuclear cells and additional staining in other cellular profiles are described according to their morphology and anatomical distribution. Morphological descriptions alone do not establish the identity of all stained cells. Foxp3 immunohistochemistry, AEC chromogen, Mayer’s haematoxylin counterstain. Scale bars: (**A**,**B**) = 100 µm; (**C–F**) = 50 µm.

**Figure 7:**
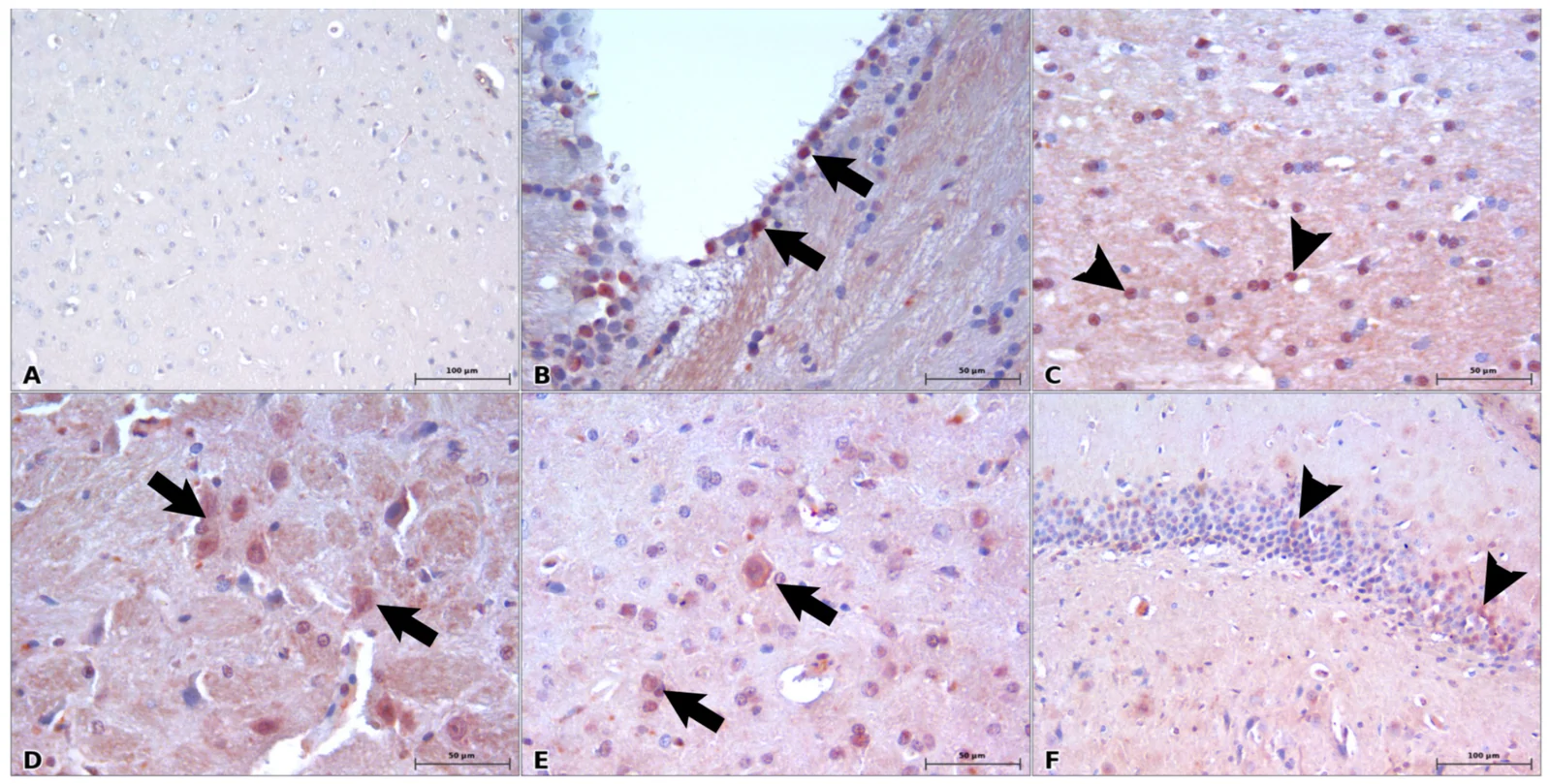
Representative temporal distribution of IL-5 immunoreactivity in the brains of control and Toxoplasma gondii-infected mice. (**A**) Control: absent or minimal immunoreactivity restricted to rare, faintly stained cellular profiles. (**B**) Day 10: a weak and focal early response characterized by cytoplasmic and perinuclear immunoreactivity in ependymal cells (arrows) and scattered cells within the adjacent periventricular parenchyma. (**C**,**D**) Day 30: pronounced multifocal granular immunoreactivity in small cells with glial or mononuclear morphology (arrow heads), the surrounding neuropil, and large polygonal or pyramidal cells (arrows) with neuronal morphology. (**E**,**F**) Day 60: more extensive and intense immunoreactivity involving large cells with neuronal morphology (arrows), smaller cells with glial morphology, the hippocampal granule cell layer (arrow heads), and the adjacent neuropil. The broader distribution in the day 60 panels is consistent with the continued increase in IL-5 immunoreactivity during the prolonged chronic phase. IL-5 immunohistochemistry, AEC chromogen, Mayer’s haematoxylin counterstain. Scale bars: (**A**,**F**) = 100 µm; (**B–E**) = 50 µm.

**Table 1:** Temporal immunoreactivity of CD68, S100B, Foxp3, and IL-5 following *Toxoplasma gondii* inoculation. Values are presented as mean ± SD. Different superscript letters within the same row indicate significant differences among groups (one-way ANOVA followed by Tukey’s HSD test, *p* < 0.05); groups sharing the same letter are not significantly different. In the Tukey HSD pattern, “<” indicates a significantly lower value, whereas “=” indicates no significant difference. η^2^, eta-squared; ω^2^, omega-squared.

Temporal Immunoreactivity of CD68, S100B, Foxp3, and IL-5 After *Toxoplasma gondii* Inoculation (Immunopositive Area (%))									
Marker	Control Group (*n* = 6)	10 DAI Group (*n* = 6)	30 DAI Group (*n* = 6)	60 DAI Group (*n* = 6)	F(3,20)	*p* Value	η^2^	ω^2^	Tukey HSD Pattern
**CD68**	1.864 ± 0.375 ^a^	6.020 ± 0.406 ^b^	8.337 ± 0.508 ^c^	8.063 ± 0.426 ^c^	288.113	*p* < 0.001	0.977	0.973	Control < 10 DAI < 30 DAI = 60 DAI
**S100B**	3.555 ± 0.452 ^a^	4.226 ± 0.496 ^a^	7.771 ± 0.506 ^b^	8.077 ± 0.349 ^b^	159.720	*p* < 0.001	0.960	0.952	Control = 10 DAI < 30 DAI = 60 DAI
**Foxp3**	0.895 ± 0.339 ^a^	1.292 ± 0.204 ^a^	4.794 ± 0.455 ^b^	5.053 ± 0.227 ^b^	285.397	*p* < 0.001	0.977	0.973	Control = 10 DAI < 30 DAI = 60 DAI
**IL-5**	1.223 ± 0.443 ^a^	1.632 ± 0.143 ^a^	4.112 ± 0.375 ^b^	4.848 ± 0.412 ^c^	146.526	*p* < 0.001	0.956	0.948	Control = 10 DAI < 30 DAI < 60 DAI
**DAI: days after inoculation**									

## Data Availability

The original contributions presented in this study are included in the article. Further inquiries can be directed to the corresponding authors.
